# Employing Evidence-Based Practices for Children with Autism in Elementary Schools

**DOI:** 10.1007/s10803-020-04706-x

**Published:** 2020-09-19

**Authors:** Ann M. Sam, Samuel L. Odom, Brianne Tomaszewski, Yolanda Perkins, Ann W. Cox

**Affiliations:** Frank Porter Graham Child Development Institute, University of North Carolina at Chapel Hill, 517 S. Greensboro Street, CB 8040, Carrboro, NC 27510 USA

**Keywords:** Evidence-based practices, Autistic students, Elementary schools, Teacher implementation

## Abstract

The purpose of this study was to test the efficacy of a comprehensive program model originally developed by the National Professional Development Center on Autism Spectrum Disorder (NPDC). Sixty elementary schools with 486 participants were randomly assigned to an NPDC and services as usual condition (SAU). Significantly greater changes in program quality occurred in the inclusive NPDC programs as compared with the SAU schools. Teachers in NPDC schools reported using more evidence-based practices (EBPs) and implemented EBPs with significantly greater fidelity than teachers in SAU schools. Autistic students in NPDC schools had significantly higher total attainment of educational goals than students in SAU schools, and the two groups made equivalent progress on standardized assessment outcomes across the school year.

The current prevalence of autism is 1 in 54 elementary-school-aged children (Maenner et al. 2020). With the average size of elementary schools now at 473 students (National Center for Education Statistics 2018), it is highly likely that there are autistic^1^ students in every elementary school in the United States. For those students, school districts must provide a free and appropriate public education (Individuals with Disability Education Act 2004) with practices based on research evidence (Every Student Succeeds Act 2015). Recent research indicates that while the overall quality of school programs for autistic students may be adequate, features of programs that focus on intervention in critical need areas may be lacking (Odom et al. 2020). Similarly, teachers often do not feel confident in providing instruction for autistic children (Van Der Steen et al. 2020). While they agree that evidence-based practices (EBPs) for students with autism and other developmental disabilities are important, they often express feeling unprepared (Knight et al. 2019). In this paper, we describe a comprehensive program model for preparing and supporting teachers to employ EBPs in their school and examine its implementation as well as its efficacy.

Autism is a neurodevelopmental impairment that begins during a child’s first 3 years and exists throughout the life cycle (Jackson and Volkmar 2019). It is defined by impairments or limitations in social communication that lead to difficulties establishing relationships and also by restrictive and repetitive behaviors that may interfere with participation in educational, home, and community settings (American Psychiatric Association 2013). From a recent epidemiological study involving over 5000, 8-year old autistic children in the United States, Maenner et al. (2020) reported that the ratio of boys to girls is about 4:1; 30% of autistic children also have intellectual disabilities; and autism occurs across race/ethnicities.

As noted, public schools must provide a free and appropriate public education that demonstrates sustained progress for students with autism. By law, school personnel must establish individualized educational goals that guide the program the school provides. Sustained progress is measured through the student’s achieving these goals. The instructional and intervention practices that teachers and other practitioners employ in addressing individualized goals must be evidence-based with some empirical demonstration of their efficacy (Individuals with Disabilities Education Act 2004). To effectively address student goals, teachers/practitioners may follow a “technical eclectic” strategy, in which they select practices that have their roots in different theoretical models and have clear evidence of efficacy (Odom et al. 2012). Teachers/practitioners select intervention practices based on their history of demonstrating positive outcomes related to student’s Individualized Education Program (IEP) goals, the characteristics and perhaps preferences of the student and/or family, and the teacher’s/practitioner’s knowledge and skill (Sam and Hume 2019). This approach is directly aligned with the process followed in evidence-based medicine (Sackett et al. 1996).

The National Professional Development Center on Autism Spectrum Disorder (NPDC) employed just such a process as the foundation for a professional development model that U.S. state education agencies employed to increase teachers’ use of EBPs (Odom et al. 2013). The model consists of four key components. First, it focused on the program quality of classrooms to enable teachers to implement EBPs because the implementation of EBPs in poor quality classes would be difficult and ineffective. NPDC investigators used the Autism Program Environment Assessment Rating Scale (Odom et al. 2018, APERS) to assess the quality and used the assessment information to establish a high quality foundation for the program. Second, teachers designed measurable and observable goals for individual students, using the Goal Attainment Scale (GAS) to establish benchmarks for student progress and goal achievement (Kiresuk and Sherman 1968). Third, teachers matched these individual student goals to EBPs that NPDC staff had identified through a systematic review (Odom et al. 2010). The final component involved teachers implementing the practices with embedded coaching by local school personnel and NPDC staff.

To assess the effects of this NPDC model, Odom et al. (2013) conducted a program evaluation of the NPDC model that was implemented in nine states. NPDC staff and local school leaders conducted training on the NPDC model for school program personnel from the nine states during the summer before an implementation year. They worked with school staff to develop observable and measurable goals for students, conducted APERS assessments, worked with school personnel to establish an action plan to improve quality, trained teachers to use EBPs linked to student goals, and coached teachers on the implementation of EBPs. In comparison with measures taken in the fall of the school year, Odom et al. (2013) found that APERS scores increased significantly, teachers reported significantly increased use of EBPs, teachers substantially increased EBP fidelity across the year, and students’ goal attainment increased significantly.

Although the NPDC program results were promising, the study was not an experimental demonstration of efficacy because it only employed a single group, and thus was open to the possible threats to internal validity. In addition, the autism intervention literature had been criticized as including primarily white children and youth as participants, with participants of color significantly under-represented (Pierce et al. 2014; West et al. 2016). The previous NDPC study did not report these variables.

The NPDC model qualifies as a class of programs called comprehensive programs (i.e., also called comprehensive treatment models). These programs are characterized by: (1) a central conceptual framework that guides program features, (2) the intensive nature of the program (e.g., more than 20 h per week), (3) program length (e.g., sustained over a school-year or more rather than weeks), and (4) focus on a variety of (rather than singular) learning or development outcomes (National Research Council 2001). Examples of these programs include the Denver Model (Rogers et al. 2000), intensive behavior intervention program based on the early program efforts of Lovaas (1971), the TEACCH program based on early pioneering work by Schopler and Reichler (1971), and the LEAP program developed by Strain and Hoyson (2000). In the original National Research Council report, most of the identified programs operated at universities or autism clinics, and the majority focused on preschool children with autism. Although the comprehensive programs have diversified since the original report (Odom et al. 2014) and some established their efficacy through RCT studies, only a few have expanded implementation in public schools (Anderson et al. 2020; Boyd et al. 2014; Strain and Bovey 2011; Suhrheinrich et al. 2020), and except for Anderson et al. they still tend to focus solely on young children.

To date, the NPDC program was unique, as a comprehensive program, in its focus on students’ goals established by teachers, other practitioners, and families as well as implementation of practices by teachers/practitioners in elementary, middle school, and high school settings. However, as noted, an efficacy trial has not yet examined NPDC effects when compared to typical services provided by schools. The purpose of the current study was to examine the efficacy of the NPDC comprehensive program when implemented in public elementary schools by teachers and other service providers. The specific research questions for this study were: (1) Can teachers implement the NPDC model in elementary schools? (2) Are there differential changes in the quality of the programs for SAU and NPDC programs? (3) Are their differences in teachers’ and service providers’ fidelity of implementation of EBPs in the SAU and NPDC groups? (4) Are their differences in autistic students’ acquisition of IEP learning goals in the NPDC and SAU groups? (5) Are there changes in standardized measures of student characteristics and/or development? (6) Are there differential outcomes related to race/ethnicity?

## Methods

All procedures were approved by the Institutional Review Board at the university where investigators worked.

### Settings

The study took place in 60 publicly funded elementary schools located in the central and eastern regions of a southeastern state in the United States. Special education schools (i.e., only enrolling students with disabilities) and charter schools were not included in this sample. School recruitment began at the district level, with district leaders approving participation in the study before schools were recruited. Research staff initially contacted 13 districts and nine agreed to participate in the study. Reason for nonparticipation were no response to initial contact (n = 1), declined to respond (3). To be eligible for the study, schools had to have at least eight students with a primary or secondary educational diagnosis of autism. At the school level, school participation was voluntary with the principal and three key school staff (e.g., resource teacher, special education teacher, general education teacher, speech language pathologist) agreeing to participate before recruitment began by signing a memorandum of understanding. The group was identified as the Autism Team (A-Team). Recruitment of schools intentionally included schools in rural, suburban, and urban areas representing a range of socioeconomic status and race/ethnicities of students to approximate demographics of the United States (Tipton 2014). Initially, research staff contacted 84 schools about participation, of which 60 initially agreed to participate. One school from the NPDC groups withdrew for the study after pretest data had been collected.

The elementary schools contained kindergarten to 5th grade classes. Schools were located in rural areas (n = 16), suburban areas (n = 18), and in cities (n = 25). The average size of schools was 620 students (see Table 1). On average, 54% of students qualified for free and reduced lunch based upon federal guidelines. Most schools had special education (58 schools) and inclusive (56) programs. In inclusive programs, autistic students spent the majority of their school day in general education classrooms and received support services provided by a resource special education teacher. In special education programs, students spent the majority of the school day in a special education class, usually with opportunities during the day to participate in classes or activities out of the separate setting (e.g., recess, lunch, physical education, art). Not all schools had both types of program settings.

**Table 1:** School demographics

Characteristics	Total (N = 60) % or M (SD)	NPDC (N = 40) % or M (SD)	SAU (N = 20) % or M (SD)	T or Χ^2^ (df)	p-value
Students receiving free and reduced lunch	54.32 (25.94)	53.93 (24.92)	55.11 (28.52)	0.16 (58)	0.87
Title 1 Eligibility	70.00	70.00	70.00	0.0 (1)	1.0
Total number of students	619.62 (204.94)	609.23 (204.48)	640.40 (209.55)	0.55 (58)	0.58
Urbanicity				0.14 (2)	0.93
City	41.67	40.00	45.00		
Suburban	31.67	32.50	30.00		
Rural	26.67	27.50	25.00		
Number of study students with ASD	8.40 (2.74)	9.05 (2.47)	7.10 (2.86)	2.73 (58)	.01
Number of study students in self-contained	5.08 (2.51)	5.55 (2.34)	4.15 (2.92)	2.10 (58)	.04
Number of study students in inclusive	3.33 (1.89)	3.50 (1.83)	3.00 (2.97)	0.96 (58)	.34

### Participants

**Fig. 1:**
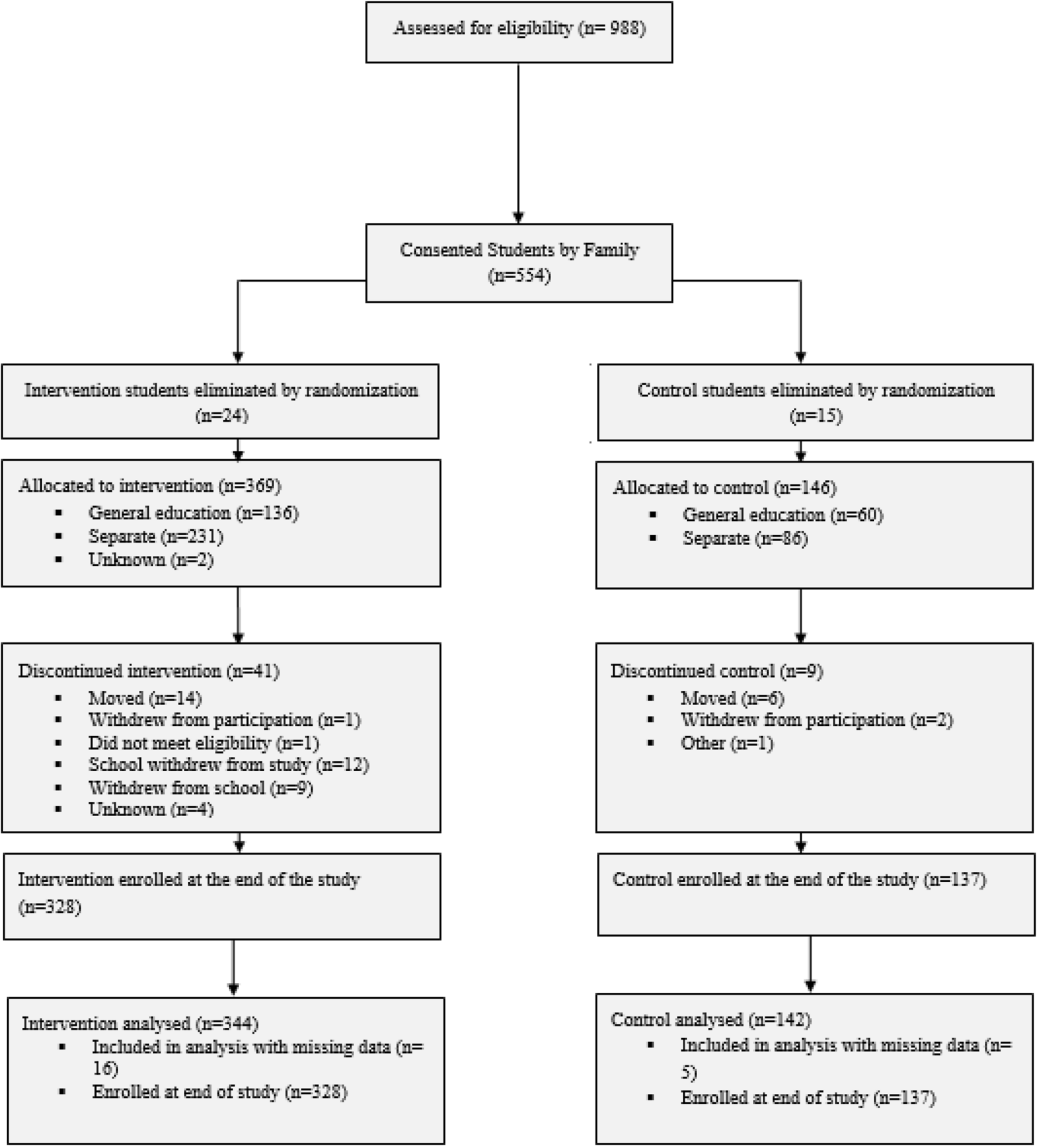
Consort table for schools with students nested

Four hundred eighty-six students participated in the study (See Consort Table in Fig. 1). Inclusion criteria were that students had a primary or secondary educational diagnosis of autism and qualified for special education services based on state guidelines. Also, a small number of students participated with an eligibility category of developmental delay and a clinical diagnosis of autism. Student participation was limited to 12 students per school. If more than 12 students returned consent forms, twelve students were randomly selected to participate in the study. The majority of students were male (see Table 2). A large portion of the sample was white (43% in the total sample), although the majority of students were non-White, Hispanic and/or multiracial. Students’ mean age was 8 years, and they were distributed across kindergarten to fifth grade.

**Table 2:** Child and family demographics

	Total		NPDC		SAU	
	N	%	N	%	N	%
*Child race and ethnicity*^1^						
Asian	31	6.4	21	6.1	10	7.1
Black	127	26.2	89	25.9	38	27.0
Hispanic^2^	85	17.5	56	16.3	29	20.6
Multiracial	34	7.0	22	6.4	12	8.5
Other	6	1.2	6	1.7	0	0
White	202	41.6	150	43.6	52	36.9
*Child gender*						
Male	382	78.6	266	77.3	116	81.7
Female	104	21.4	78	22.7	26	18.3
*Child grade*						
Kindergarten	71	14.6	53	15.4	18	12.8
1st	90	18.6	59	17.2	31	22.0
2nd	95	19.6	69	20.1	26	18.4
3rd	79	16.2	53	15.4	23	18.4
4th	76	15.7	53	15.4	23	16.3
5th	70	14.4	55	16.0	15	10.1
Other	4	0.8	2	0.6	2	1.4

	M	SD	M	SD	M	SD
*Child age*	8.35	1.80	8.40	1.83	8.22	1.70
*Estimate of annual household income*^3^	58,020	23,882	58,533	24,558	57,012	22,200
*Nonverbal IQ*	79.31	24.51	78.91	24.89	80.29	23.61
*Adaptive behavior ABC*	68.60	17.12	68.72	17.40	68.32	16.46
*Social Communication Questionnaire Lifetime*	20.97	7.16	20.97	7.03	20.99	7.52

^1^No report of race/ethnicity for one student

^2^Self identified as White/Hispanic

^3^Annual household income estimated from families address and census information because of degree of missing data from parent self-report

Specific school staff members participated on the A-Team, although other nonA-team school staff members also completed student assessments and/or addressed identified student goals. The A-Team consisted of special education teachers, general education teachers, administrators, speech language pathologist, and others, and the majority were white, nonHispanic, and women. An NPDC coach was assigned to all schools. All coaches had at least an undergraduate degree in education or psychology and had worked with students with autism as a teacher, a district-level coach, or clinician. Coaches received extensive training on the NPDC model before beginning to work with schools and subsequent supervision from research investigators.

### Experimental Conditions

#### Randomization

Investigators randomly assigned schools to an NPDC and services as usual (SAU) condition following a 2 (NPDC) to 1 (SAU) ratio. When possible, randomization occurred within districts. If this was not possible (e.g., < 3 schools in a district) schools were matched on school demographic and geographic characteristics and randomly assigned in groups of three.

#### NPDC

In the summer before NPDC was to be implemented, investigators conducted a 1-day training academy for the school A-Teams. The training academy provided an orientation to the NPDC program model (e.g., included information about the APERS, an overview of evidence-based practices, the selection of evidence-based practices to address an identified student goal, a review of two evidence-based practices, and the NPDC coaching process). During the training academy, all team members received an online Introduction to Autism overview. Additionally, the A-Team participated in a workshop on the GAS, which involved an introduction of GAS, components of a measurable goal (antecedent, behavior, and criteria), description of how to scale the goal across levels, and quality indicators for GAS (i.e., measurability, difficulty, equidistance).

Within the first 10-weeks of school, an APERS was conducted by a NPDC research staff member not associated with the school being assessed. The APERS included 6-h of observation, record review, and interviews of key staff members. A report was provided to A-team members, which described strengths, areas of needs, and suggestions for the next steps to improving program quality. Based on the report, the A-Team developed a school plan with a suggested two areas of need to be addressed based upon the APERS report. The A-Team met with the NPDC coach four times during the school year to address the school plan.

For each student participant, A-Team members, other school personnel, and NPDC coaches selected three goals from students’ IEPs. For each goal, the group followed the Psychometric Equivalence Tested-Goal Attainment Scale (PET-GAS; Ruble et al. 2012), described in detail in the next section. Once the goals were scaled, teachers selected, with coaches’ assistance, an EBP that would address each students’ goals. The specific EBP was chosen from the 27 focused intervention practices that Wong et al. (2015) had identified as having evidence of efficacy. Online learning modules with observational fidelity checklists had been created for each of the EBPs and were available through the Autism Focused Intervention Materials and Resources website (AFIRM, Sam et al. 2020; https://afirm.fpg.unc.edu/node/137). Child characteristics, teacher characteristics, and resources were then discussed to determine the most appropriate EBP to address the identified goal (see https://afirm.fpg.unc.edu/selecting-ebp for a detailed description of the EBP selection process). When a teacher selected an EBP, they were introduced to the corresponding AFIRM online learning module, received coaching on using the EBP (described in next paragraph), and received weekly performance feedback on the fidelity of the use of the EBP until they reached the fidelity criterion of 80% or higher.

NPDC coaches were expected to spend 6 h a week at each school. Coaches followed the NPDC model of pre-observation meetings, observations, and post-observation debriefs to coach on selected EBPs (Kucharczyk et al. 2012). Pre-observations involved coaches reviewing implementation practices of the fidelity of selected practices and how the staff member would implement the practice with the selected student. During observations, coaches completed the implementation checklist of the staff member to determine the fidelity of the selected EBP by the staff member. During the debrief, the implementation checklist was reviewed by the coach, and the next steps were determined to increase fidelity.

#### Services as Usual (SAU)

For the SAU schools, an A-Team was formed (before randomization). The NPDC research staff gave A-Team and other school personnel access to the online Introduction to Autism overview, with their completing the module being optional. Additionally, A-team members received a ½ day training that included an orientation to the research project as well as PET-GAS training and scale development. Research staff not associated with the school conducted an APERS at the SAU schools within the first 10 weeks of the start of the school year. Research staff shared a report of the results with the A-Team, but research staff did not assist the team in developing a school plan based upon results. After pre-test data collection, research staff checked in via email periodically with members of the A-Team (approximately 2–3 times a year) to see if team members had questions or concerns.

### Implementation Index

To assess the implementation of the NPDC model, investigators developed an implementation index following the procedure established by Steinbrenner et al. (2020). The index consisted of six components: (1) A-Team formation, (2) Participation on A-Team, (3) Professional Development for A-Team, (4) Program Quality Activities, (5) GAS Goal Development, and (6) Evidence-based Practices. The implementation index consisted of a three-point rating scale (i.e., 1 = incomplete, 2 = partially complete, 3 = complete) for items grouped within each component. Data for each item were collected during the school year from all schools, and investigators used those data to completed implementation ratings after post-tests. The implementation index demonstrated high internal consistency (Cronbach’s α = 0.88).

### Assessing Program Quality

As noted, project staff assessed the quality of school programs using the preschool elementary version of the APERS (*APERS-PE*) (Odom et al. 2018). The *APERS-PE* is a 56-item rating scale consisting of 10 domains (learning environment, positive learning climate, assessment and IEP development, curriculum and instruction, communication, social competence, personal independence and competence, functional behavior, family involvement, and teaming). Items are based on a five-point Likert-type rating continuum. The “1” rating indicates ***poor*** quality, the “3” rating indicates acceptable quality, and the “5” item represents excellent quality. Research staff (i.e., never the coach working at the school) conducted observations in the school over 2 days, interviewed school staff (e.g., special education teachers, general education teachers, related services personnel, principal) and two family members, and analyzed documents (e.g., IEPs). They then used these information sources to complete the ratings. The APERS-PE was collected at the beginning and end of the school year. Separate assessments ratings were completed for the Special Education and Inclusive programs. For schools that had both programs, a weighted APERS-PE was calculated based on the proportion of autistic students enrolled in each program in the school.

To check the interrater agreement, a second rater completed the APERS-PE for 20% of the schools. Interrater agreement was calculated at the item level. Average inter-rater agreement across items that were an exact match was 65.9%, and 87% were within one rating point. Interrater Reliability was calculated using Intraclass Correlations (ICCs) for the total APERS-PE items. There were high ICCs for the overall totals (.97 and .98 for the special education and inclusive programs, respectively). Cronbach’s alphas were calculated as a measure of internal consistency and were high for the overall totals (.93 and .96).

### Dependent Variables

#### Teacher Attitude Toward, Use of and Fidelity of EPBs

Research staff collected information on school staff attitude toward, use of, and fidelity of EBPs. To assess teachers’ attitudes about the use of evidence-based practices, teachers completed the 15-item teacher version of the Evidence-Based Practice Attitude Scale (EBPAS, Aarons et al. 2007). The EBPAS results in a total score and scores for four subscales: *Appeal* (extent teacher would adopt an appealing EBP), *Requirements* (extent teacher would adopt an EBP if required to do so), *Openness* (the extent to which the teacher is willing to try new EBPs), and *Divergence* (the extent to which the teacher views EBP as less important and not useful). Also, teachers on the A-Team completed the Evidence-Based Practice inventory (EBPI), which lists all 27 EBPs from Wong et al. (2015) and asks teachers to rate whether they used the practice in their classroom very often (3 rating), sometimes (2 rating), or not at all (1 rating).

Research staff and coaches collected fidelity on teachers’ use of each EBP using the implementation checklists from the AFIRM modules. The checklists consist of 10–21 items, and the coach/staff records a yes or no for each item. The fidelity metric is the percentage of items with a yes rating. For NPDC schools, the coach completed the fidelity measure checklist during observations with school staff. If a teacher obtained 80% fidelity or higher, the coach then observed the teacher three more times if time allowed in the school year, to assess maintenance of fidelity without coaching. For SAU schools, teachers identified the EBPs they were using in their class, through the EBPI, and the students with whom they were using the EBP to address student IEP goals. Research staff completed fidelity checks on 20% of EBP identified by school staff at SAU schools.

#### GAS

The GAS was used to rate student progress towards the selected goal on a 0–4 scale. A score of 0 indicates baseline performance, 1 = meets the initial objective, 2 = meets the secondary objective, 3 = attainment of the goal, and 4 = progress greater than expected see. In collaboration with research staff, teachers established three GAS goals per student and completed the GAS rating at the end of the school year. For 26% of the GAS ratings at the beginning of the year (baseline) and end of the year (post-test), research staff also rated the student goals based on their observations and data collection. The research staff and teachers had an exact agreement on student goal level for 85.8%.

To establish the measurement quality of the goals written, the Psychometric Equivalence Tested-Goal Attainment Scale process (PET; Ruble et al. 2012) was used to establish the quality of the goals developed. The PET evaluates the steps between rating items (of individual goals) based on measurability (e.g., observable, quantified), difficulty (e.g., the progression from current to more advanced performance), and equidistance (e.g., a similar amount of progress between rating steps). Research staff assigned a score of 1 (not at all difficult/measurable/equal) to 3 (very difficult/measurable/equal) in each of the psychometric quality dimensions for all goals (n = 1461). Inter-rater agreement data were collected on 63% of the PET evaluations. Research staff agreed on 91% of the PET scores (i.e., equivalent for NPDC and SAU goals). Mean ratings were 2.18 for Difficulty (NPDC = 2.20, SAU = 2.15), 2.52 for measurability (NPDC = 2.52, SAU = 2.15) and 2.38 for equidistance (NPDC = 2.42, SAU = 2.28). None of these ratings were significantly different for NPDC and SAU groups, and all were in the range of psychometric equivalence that Ruble et al. (2012) reported in their initial article. See Table 3 for more details.

**Table 3:** PET-GAS scores

PET-GAS domain	NPDC (N = 39)				SAU (N = 20)			
	M	SD	Min	Max	M	SD	Min	Max
Difficulty	2.20	0.32	2.09	2.30	2.15	0.23	2.04	2.26
Measurability	2.61	0.45	2.46	2.75	2.35	0.50	2.12	2.49
Equidistance	2.42	0.55	2.24	2.61	2.28	0.57	2.02	2.55
Total goals	26.54	7.27	9	36	21.30	7.96	9	36

### Standardized Norm-Referenced Measures

At pre-test and post-test teachers and school staff completed the Social Skills Improvement System (Gresham and Elliott 2008), Children’s Communication Checklist-2 (Bishop 2006), Repetitive Behavior Scale (Lam and Aman 2007), Vineland Adaptive Behavior Scale-II Teacher Form (Sparrow et al. 2006), and Academic Performance Rating Scale (DuPaul et al. 1991). Parents included Social Communication Questionnaire-Lifetime (Rutter et al. 2003), and a demographics form. Additionally, the Leiter-3 was used at the pre-test to determine the nonverbal measure of intelligence (Roid et al. 2013).

### Statistical Analysis

The statistical analysis followed an intent-to-treat analysis with general linear models (e.g., ANOVAs and independent t-tests) to examine school outcomes (i.e., Implementation Index, APERS-PE, demographics) and 2-level or 3-level hierarchical linear models to account for teachers and students nested in schools when examining teacher and student outcomes (i.e., GAS scores, standardized measures). The Time × Intervention Group interaction represents the difference in change in outcomes between NPDC and SAU groups. Hedges’ *g* effect sizes are reported for these effects. All analyses were performed with SAS PROC MIXED Version 9.4 (SAS Institute, 2016), restricted maximum-likelihood estimation, and Satterthwaite or Kenward-Roger approximations to determine degrees of freedom. We corrected for multiple comparisons using the Benjamini–Hochberg procedures (Benjamini and Hochberg 1995) within the sets of analyses when applicable. To examine the race/ethnicity research question, the statistical models for student-level outcomes were expanded to include race variables representing six race and ethnicity groups (Asian, Black, Hispanic, Multiracial, Other, and White) and their interactions with the intervention group for the GAS outcomes. Three-way interactions provided estimates of whether condition effects varied by racial group.

## Results

### Randomization

As noted, schools were the unit of randomization for this study, and students were nested within schools. Chi-square or T-tests were performed to examine differences between NPDC and SAU groups on school, student, and school staff characteristics at pretest. There were no significant differences between NPDC and SAU students on age, grade level, nonverbal IQ, race/ethnicity, gender, parental education, or household income. Similarly, there were no significant differences in school and A-Team staff characteristics, with the exception that there were significantly more students in NPDC schools.

Because this study took place in a single southeastern state, a question exists about the generalizability of the findings. Using the school data, the investigators employed the Generalization Index to determine the degree to which the findings of this study could be generalized to a broader population sample (Tipton et al. n.d.). The generalizability index assesses the degree to which a sample is representative of an inference population, which for this study is the United States (Tipton 2014). Scores range between 0 and 1 with scores in the 1–0.90 range categorized as *very high*, 0.90–0.80 as *high*, for scores between 0.80–0.50 as *medium*, and scores below 0.50 as *low*. The schools in this study sample were very highly representative, with a score of 0.92 based on the generalizability index.

### Implementation Index

The degree of implementation for NPDC and SAU schools, as measured by the implementation index, is found in Table 4. The range of the scores is from 1 to 3. The mean item rating for NPDC was 2.49 (out of 3), indicating that the NPDC schools were generally implementing the intervention as planned, and their implementation was significantly different from SAU schools (i.e., rated at 1.69). Investigators employed independent samples t-tests to analyze the difference between the NPDC and SAU schools, finding significant differences (i.e., with Benjamini–Hochberg corrections) for all but the A-Team variable (p < .06), which was slightly over the .05 level but with Hedges’ *g* standardized effect size of .65). In fact, for the SAU schools, an A-Team was formed and served as the point of contact between the schools and the research project.

**Table 4:** Implementation Index Scores between NPDC and SAU schools

Domain	NPDC	SAU	t	p-value	Hedges’ *g*
	M (SD)	M (SD)			
A-Team	2.73 (0.45)	2.35 (0.78)	2.01	.055	0.65
Participation on A-Team	2.60 (0.43)	1.62 (0.27)	10.65	< .001	2.55
Professional development	1.97 (0.49)	1.35 (0.38)	4.87	< .001	1.36
Program quality	2.80 (0.29)	1.94 (0.16)	14.82	< .001	3.38
Student outcomes	2.16 (0.36)	1.85 (0.38)	3.08	.003	0.87
Using evidence-based practices	2.71 (0.32)	1.05 (0.15)	22.08	< .001	6.03
Overall	2.49 (0.20)	1.69 (0.13)	16.33	< .001	4.45

### APER-PE

**Fig. 2:**
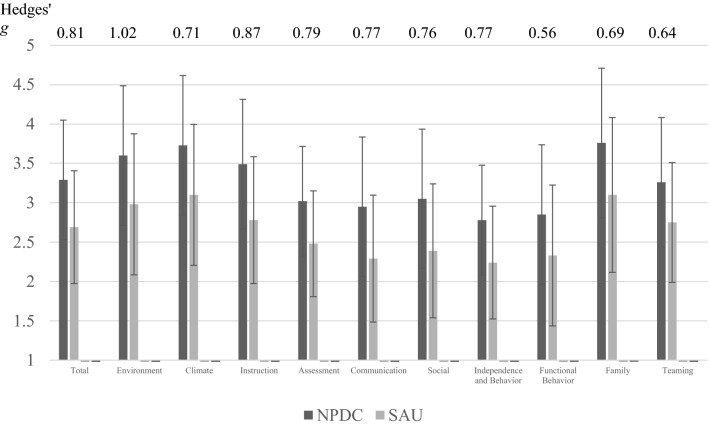
Adjusted post-test mean item ratings with standard deviations for APERS-PE and Hedges’ g

A repeated measures ANOVA was performed to examine changes from APERS-PE Total Weighted Score at Pre to Post between NPDC and SAU schools. The Intervention Group effect was statistically significant, Estimate = − 0.60 SE = 0.12, t (58) = 2.91, *p* = .005, indicating that the SAU group had a significantly lower school quality score at pretest than NPDC schools, despite randomization. The Time effect was statistically significant, Estimate = .33, SE = .09, t(57.4) = 3.90, *p* < .001, indicating that both groups made significant gains in overall school quality from pre-test to post-test. However, the Time × Intervention Group was not statistically significant, suggesting that while the NPDC group did make gains, they did not make more significant gains in school quality from pretest to posttest than the SAU group. Repeated measures ANOVAs were performed across the ten APERS domains, and there were no statistically significant interactions using a Benjamini–Hochberg procedure to correct for multiple comparisons. Effect sizes were calculated between NPDC and SAU schools for post-test means adjusted for pre-test means. These adjusted posttest means appear in Fig. 2, were substantially higher for the total score and each of the domains, with substantial Hedges’ g ranging from 0.56 to 1.02.

The substantial effect sizes for the weighted APERS-PE scores favoring the NPDC sample led to a further examination of the APERS-PE data in which the special education and inclusive APERS-PE findings were examined separately. Repeated measures ANOVAs detected a significant Group × Time interaction effect for the inclusive program, F (1,51.8) = 7.41, *p* = .01 for the APERS-PE Total Mean Item rating score. Also, for the APERS Social domain there was also a significant interaction effect (F (3,105) = 6.77, *p* = .01. There were no significant Group × Time interactions for special education programs.

### Teacher EBP Fidelity

**Fig. 3:**
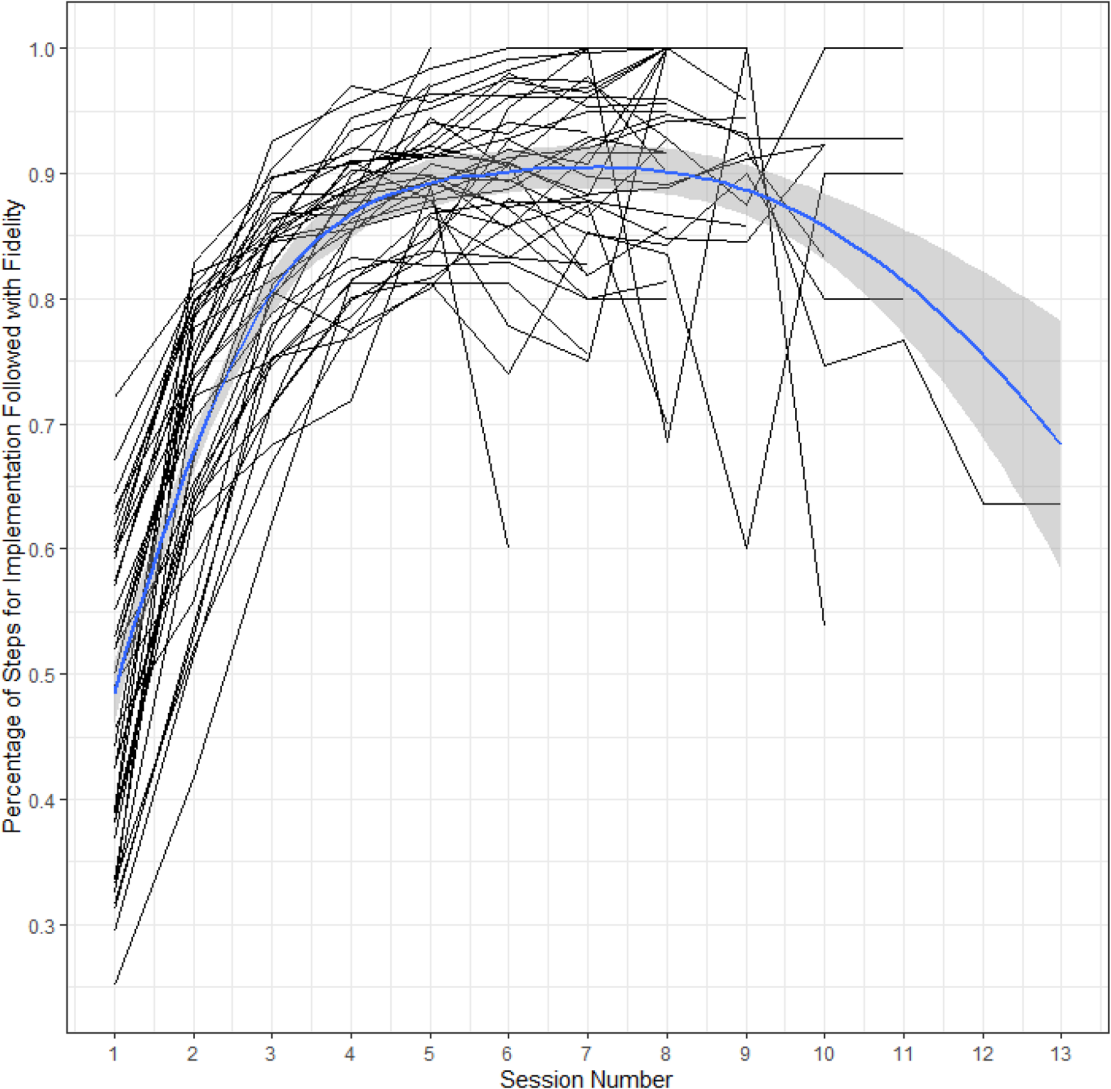
School average of teacher percentage of steps correct for implementation for each session with best fitting Line for 39 NPDC schools

NPDC coaches collected fidelity of implementation data probes for teachers in the NPDC group and individual probes for the teachers in the SAU schools. A 2-level HLM analysis was performed to examine differences between teacher fidelity scores in NDPC and SAU schools. For all teachers in the NPDC condition, the mean fidelity rate was 86%, as compared to 50% for teachers in SAU schools, which was a significant difference between groups, t (201) = 12.31, *p* < .001, Hedges’ *g* = 1.69. In Fig. 3, the mean percentage of correct fidelity steps, from the fidelity checklists, is graphed by NPDC teachers (i.e., mean fidelity scores per session) and across sessions. The criterion set for successful implementation was 80%. The graph was made using the R package ggPlot2 using the “gam” smoothing method and formula = y ~ s(x, bs = “cs”). The display for 241 teachers revealed that generally, teachers reached fidelity after 3–5 sessions. However, for some teachers, the fidelity sessions were extended beyond that time point, and a few never reached fidelity. This latter point resulted in the negative trend in the curve as the number of sessions extended. For the NPDC teachers, maintenance fidelity probes were also collected (not graphed), generating a mean fidelity percentage across maintenance sessions of 68% (SD = 28%).

### Teacher Attitudes Toward EBPs

As noted, teachers on the A-Team in both groups completed the EBPAS at pretest and posttest. A 2-level HLM analysis detected a group × time interaction for the Appeal domain of the EBPAS, F (1, 408) = 6.87, *p* = .009, adjusted Benjamini-Hochberg *p-*value = .04, Hedges’ *g* = .18 favoring the NPDC teachers. Significant differences were not found on other EBPAS subscales.

### Teacher Use of EBPs and Confidence

A set of 2-Level HLMs were performed, examining teachers’ use of EBPs and their confidence in using the identified EBPs. There was a significant time × group interaction effect for teachers’ frequency of use, F (1,703) = 7.98, *p* = .005, and teachers’ confidence in using evidence-based practices, F(1, 701) = 7.08, *p* = .005. For both analyses, NPDC teachers made greater gains than SAU teachers from pre to post.

### GAS

The mean GAS total score and scores for subdomains are found in Table 5. A series of 2-level HLMs were performed with students nested in schools to examine the GAS total scores at post-test and also scores for the Academic, Social, Communication, and Social Readiness domains. A significant effect occurred for the total GAS scores, F(1,57) = 24.37, p < .001, with students in NPDC schools having higher scores than students in SAU schools. Similar significant effects occurred for Academic, F(1,57) = 10.96, *p* = .002, adjusted Benjamini–Hochberg *p-*value = .003, and Communication, F(1,53) = 18.12, *p* < .001, adjusted Benjamini–Hochberg *p-*value < .001, domains..

**Table 5:** GAS scores for NPDC and SAU students

Domain	NPDC		SAU		Hedges’ *g*
	N	M (SD)	N	M (SD)	
Total goals**	328	2.70 (0.8)	137	2.07 (0.9)	0.70
Academic**	242	2.62 (1.1)	113	2.05 (1.1)	0.52
Social	135	2.72 (1.1)	38	2.30 (1.0)	0.38
Communication*	148	2.69 (1.1)	72	1.78 (1.1)	0.83
School readiness	81	2.84 (1.0)	23	2.43 (1.3)	0.38
Other	29	2.40 (1.3)	13	2.30 (1.1)	0.08

*p < .003

**p < .001

### Standardized Norm-Referenced Measures

Mean for standardized norm-referenced measures are displayed in Table 6. A set of 2-Level HLMs with students nested within schools and repeated measures were conducted for all domains for standardized assessments. Although there were no group main effects, there was significant change across time for both groups on the Vineland, several subscales of the SSIS, the CCCS2, and the subscales of the APRS. There were no significant Group × Time interactions for any of the measures.

**Table 6:** Standardized assessments pre-post scores and significant time main effects

Measure	NPDC		SAU		Significant main effects of time		
	Pre M (SD)	Post M (SD)	Pre M (SD)	Post M (SD)	F-value	p-value	Adjusted p-value
VABSII Communication SS	70.1 (17.9)	72.9 (19.5)	71.1 (17.2)	72.4 (18.0)	19.47	< .001	< .001
VABSII Daily Living Skills SS	70.2 (19.2)	73.3 (21.0)	72.0 (18.5)	73.6 (17.7)	16.38	< .001	< .001
VABSII Socialization SS	69.4 (14.8)	72.4 (16.1)	71.3 (14.6)	73.8 (15.8)	31.52	< .001	< .001
SSiS Social Skills SS	75.4 (17.2)	79.4 (18.0)	77.0 (18.4)	79.2 (17.7)	36.52	< .001	< .001
SSiS Problem Behaviors SS	113.2 (12.8)	112.7 (13.0)	112.7 (12.7)	111.6 (12.7)	*ns*	*ns*	*ns*
SSiS Academic Competence SS	86.6 (15.3)	88.2 (15.5)	85.7 (14.7)	89.3 (15.3)	20.70	< .001	< .001
RBSR Overall Score	0.5 (0.4)	0.5 (0.4)	0.5 (0.4)	0.6 (0.4)	4.23	.04	.05
CCC2 General Communication	77.0 (12.9)	78.2 (14.0)	74.5 (13.0)	77.5 (13.9)	8.76	.01	.01
SCQL Total	21.0 (7.0)	20.5 (6.8)	21.0 (7.5)	22.6 (7.4)	*ns*	*ns*	*ns*
APRS Academic Success	17.4 (6.8)	19.0 (7.2)	17.2 (6.7)	18.6 (6.7)	42.28	< .001	< .001
APRS Impulse Control	8.1 (2.3)	8.5 (2.4)	8.3 (2.2)	8.3 (2.2)	*ns*	*ns*	*ns*
APRS Academic Productivity	34.0 (9.3)	36.5 (9.6)	34.1 (9.0)	35.5 (8.8)	29.27	< .001	< .001

### Race/Ethnicity Analysis

On the GAS student outcomes, there was a statistically significant interaction between the intervention group and Black students for the Independence and Behavior Scores. Black students in the SAU group (M = 1.65, SE = 0.24) had a significantly lower GAS Independence and Behavior scores than White students in the SAU group (M = 2.63, SE = 0.21, Mean Difference = 0.98, t (278) = − 3.28, *p* = .001, Hedges’ *g* = 0.32). NPDC Black (M = 2.89, SE = 0.15) and White students (M = 2.86, SE = 0.12) did not have a statistically significant difference in their Independence and Behavior Scores.

**Fig. 4:**
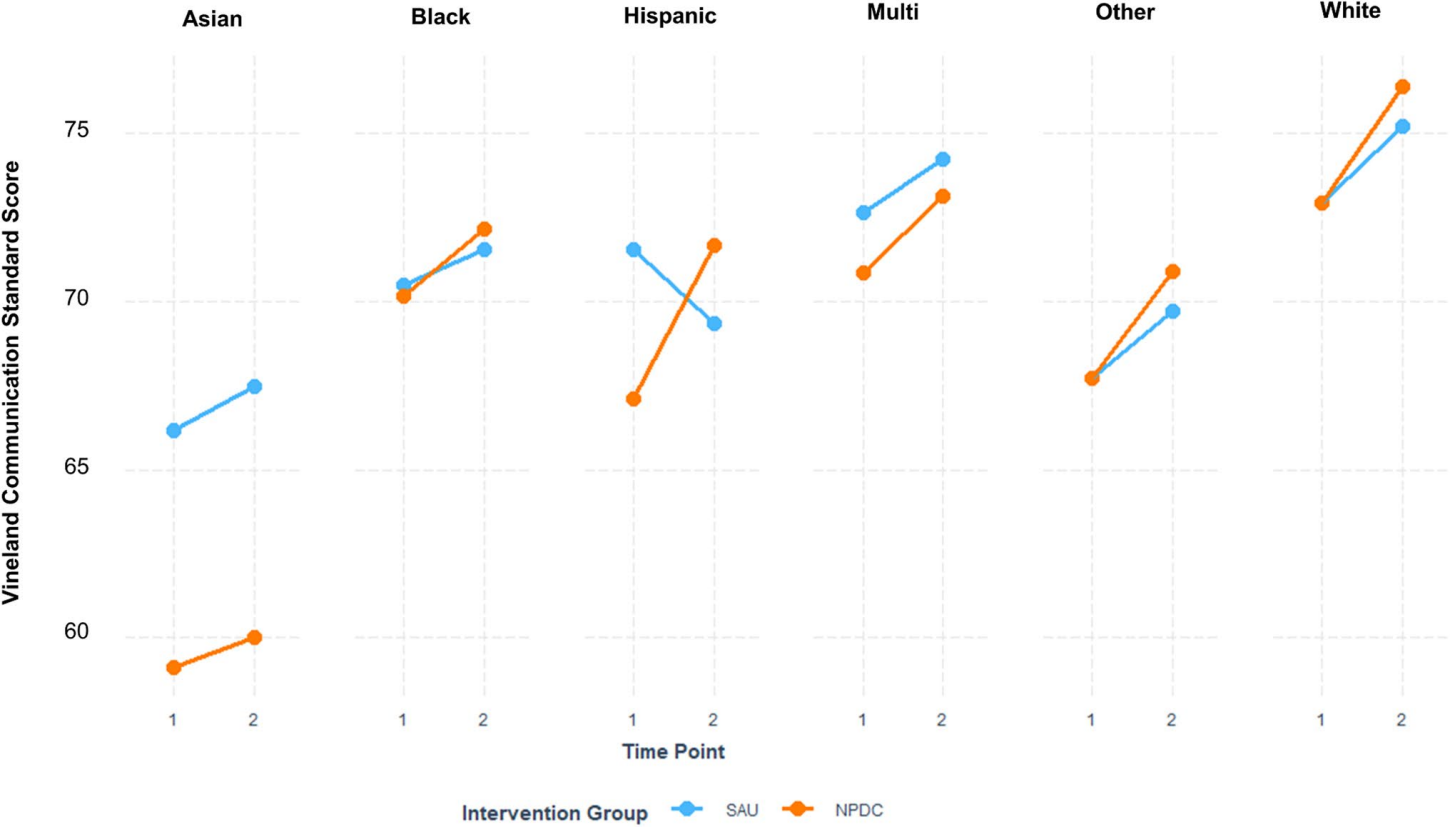
Vineland Communication Standard Scores by race/ethnicity groups by intervention group and time point

On the student standardized assessments, differential gains by condition were greater for Latinx students compared to White students on the Vineland Communication Standard Score (*p* = .02; See Fig. 4). Whereas Latinx students in the SAU schools decreased their average Communication Standard Scores over time (− 2.34 points, M = 68.94, SE = 3.62), Latinx students in the NPDC group increased their Communication scores (+ 4.59 points, M = 70.98, SE = 2.58). White students in both the control group and intervention group increased slightly in their average Communication score (NPDC =  + 3.56 points, M = 75.92, SE = 1.72, SAU =  + 1.94 points, M = 74.95, SE = 2.82).

## Discussion

The purpose of this study was to examine the efficacy of the NPDC professional development model for promoting teachers’ use of EPBs with autistic students enrolled in elementary school special education and inclusive programs. This comprehensive program differs from more individually focused (e.g., EIBI programs, clinic-based programs) and other school-based programs. In individually focused programs, the emphasis is on a therapist delivering the intervention to individual children and/or families in clinic and sometimes home settings (e.g., Early Start Denver Model). In most school-based intervention programs, researchers tend to deliver interventions in individual classrooms (CPRT). As delivered in the current study, the NPDC intervention was delivered by multiple providers (e.g., teachers, paraprofessionals) to autistic children in different school contexts. Although this breadth limits our asking more individual-oriented questions (e.g., do students with certain autism characteristics respond differently to the program), it does represent the authentic circumstances that school-districts leaders face when providing intervention services to their autistic students. Certainly, questions related to phenotype and response to intervention in school settings could be the focus of future research.

Given the multi-component, “school-wide” nature of the program, to assess implementation, investigators employed an index approach previously employed in an investigation of a comprehensive program for high school students with autism (Steinbrenner et al. 2020). The results indicated that the index assessment had a high level of internal consistency. Schools in the NPDC condition had significantly higher index ratings compared to SAU school, indicating that experimentally the program was delivered as planned and also psychometrically, the instrument displayed significant criterion-related validity (Nunnally and Bernstein 1994). Of note, the one subscale on which the NPDC and SAU schools did not differ was on the formation of an A-Team, which was in alignment with expectations in that A-Teams were to be established at schools in both groups (i.e., in NPDC to implement the intervention and in SAU to serve as school contacts with the research project). Many comprehensive programs have been employed with students having autism, but in fully-powered efficacy studies there have been few attempts to assess the comprehensive, multi-component implementation of such programs (Odom et al. 2014).

The NPDC model addresses program quality as the foundation on which to base teacher utilization of EBPs and used the APERS-PE to assess quality. Although findings on the weighted APERS-PE were not significantly different, the effect sizes were substantial, so investigators probed further by analyzing the APERS-PE separately for inclusive and special education programs. The NPDC model appeared to impact the inclusive program the most; in that, a significant difference was found for the total ratings in inclusive programs but not for the special education classes. Also, for inclusive programs, ratings were significantly higher for NPDC for the social competence subdomain. It could well be that training provided to general education teachers and resource teachers created the possibility for higher quality social milieu reflected in the social competence domain scores. These findings partially replicate the results of a study by Hume et al. (2020) in which a comprehensive autism program at the high school level had a significant impact on program quality. Few programs to date have examined overall changes in program quality that result from comprehensive autism program models implemented in schools, which could be a direction for future research.

A second research question was about the teachers’ use of EBPs. Teachers in NPDC classrooms reported significantly higher numbers of EBPs used in their program and greater confidence in using the EBPs, as compared to teachers in the SAU programs. In addition, teachers in the NPDC classroom reported significant increases in the appeal of EBPs for working with students having autism. The findings were aligned directly with the primary goal of the NPDC program, which was to increase teachers’ use of EBP practices and replicate the results of the Odom et al. (2013) evaluation study experimentally. The program addressed positively the main concern that some teachers have with the absence of training available and their lack of confidence in using EBPs (Knight et al. 2019).

Although reported use of EPBs is important, the teachers’ use of the EBPs with fidelity is the second dimension of EBP use that is essential. In this study, coaches provided training and performance feedback on teacher fidelity. The data graphed in Fig. 2 illustrated relatively lower levels of fidelity during the initial use of the EBP and sustained increases in fidelity over the first 3–5 sessions. The findings of this study blend with a growing literature indicating that initial training, in-school coaching, and performance feedback can lead to increases in fidelity (Rosenberg et al. 2020; Snyder et al. 2015). However, the data also revealed that some teachers needed additional sessions to reach fidelity, which suggests that in future research models of professional development and coaching may need to adopt an adaptive design (Kai et al. 2020) in which different levels of support are provided to teachers based on the progress they make in learning and implementing specific EBPs.

For schools and teachers, the primary objective is for students to achieve their educational learning goals, as operationalized on their IEPs. In this study, investigators used the GAS as a primary measure of student progress. They found that the measure met the standard for “psychometric equivalence” as identify by Ruble et al. (2012) and that teachers could provide a rating that was reliable with the research staff’s rating of student progress. Students in the NPDC schools made significantly greater gains on total GAS goals and on goals in the communication and academic domains that students in the SAU schools. These data experimentally replicate findings from the previous Odom et al. (2013) program evaluation study.

Investigators found significant changes from the beginning of the year to the end of the year for students in both the NPDC and SAU groups on a battery of standardized norm-referenced measures, but no group by time interactions. Public school programs focus on student educational goals that are individually determined based on student needs, skills needed for independence and success in current and future environments, and family priorities. As such, norm-referenced standardized scores are less relevant in school-based programs than is progress on individualized goals. In contrast, some comprehensive autism programs have explicitly focused on changing the core features of autism (Haglund et al. 2020), noting that IEP goals are not always aligned with these core features (Anderson et al. 2020). In such cases, changes in diagnostic (e.g., ADOS) and standardized norm-referenced measures (e.g., SRS, SCQ) could be considered the primary outcome variables of interest. However, Pellecchia et al. (2020) have noted the importance of monitoring students’ performance on such distal outcomes to ensure that the implementation of an intervention programs does not disrupt the broader development of students in classrooms. The significant cross-time effects for both groups indicated that NPDC could be implemented without negatively affecting student performance on these distal measures.

Responding to criticisms of the lack of reporting and analysis of race/ethnicity in the autism intervention literature (West et al. 2016; Pierce et al. 2014), in this study investigators examined outcomes for Black, Hispanic, Asian, mixed-race, and white students separately. For Black students, goal attainment for independence and behavior was significantly lower than for white students in the SAU schools. At the same time, the patterns were not found in the NPDC schools where the progress was nearly equivalent. For Hispanic students, the pattern of communication growth as reflected in the Vineland Communication subscale across the school year was of concern in that Hispanic students in the SAU group appeared to regress while Hispanic student in NPDC classes made progress. These findings suggest that for some outcomes black and Latinx students with autism may benefit less from special education services that white children with autism, and the NPDC model appeared to counter such negative effects in some instances. This study also illustrates the importance of “disaggregating” findings to examine differential patterns of performance associated with race/ethnicity. Low numbers of students from different races/ethnicities in study samples reduce power and make such analyses difficult, but they can still be explored to identify suggestive patterns of performance. In our diverse society, it is no longer plausible to ignore the possibility of differential response to intervention (or to even the standard practices in SAU schools) related to race/ethnicity.

Limitations also exist for this study. First, we were unable to utilize data collectors who were blind to the experimental condition of schools, with research staff and teachers being the primary data collectors and informants for rating scales. These could have introduced bias into the dependent variable. Future studies would benefit greatly from incorporating naïve data collectors. Also, with regard to measurement, we acknowledge that the Vineland Adaptive Behavior Scale-2 has been criticized for having some items that are culturally biased (Manohari et al. 2013; Taverna et al. 2011), so findings from the Vineland should be interpreted with caution. Also, our sample of schools were not randomly selected for the larger set of school in the state, but rather had agreed to participate in this study before randomization. This could reduce generalizability to other contexts, although we did find using the Generalizer that the characteristics of the schools approximated a nationally representative sample. Last, using the term SAU is a bit of a misnomer in that the SAU school staff did receive NPDC materials and information about their school’s program quality. The features that differentiated the two conditions were the more intense and targeted initial training, coaching, and performance feedback. These findings are consistent with other efficacy studies of comprehensive programs in which simply providing a workshop and materials along was not sufficient to produce effects when compared with a condition in which ongoing coaching and feedback is provided (Strain and Bovey 2011).

In conclusion, NPDC appears to be a comprehensive program model that can be implemented in public schools with coaching assistance and increases teachers’ use of EBPs with fidelity. It did not disrupt the developmental progression of autistic students enrolled and had a positive impact on autistic students’ attainment of individualized learning goals. The study validates NPDC as an option for school districts and practitioners who are searching for ways to promote EBP use in their programs.

## Footnotes

1 In this document, we will use a mixture of terminology when referring to autism and persons identified as autistic. A common form of description has been called “person-first”, in which the person (e.g., child) appears before the condition (e.g., autism), such as child with autism. Many autistic self-advocates and advocacy groups now prefer an identify-first form, such as autistic child (Brown 2011; Kenny et al. 2016). In addition, autistic advocates have spoken about the desirability of using the term “autism” rather than autism spectrum disorder (Brown 2011). At the time of this writing, terminological issues have not been settled. To honor the advocates and professionals in the field, as well as other groups of individuals with disabilities who prefer the person-first term, we will be mixing terminology throughout the manuscript, using both person-first and identity-first terminology with the primary descriptor being autism or autistic.
